# Quantitative Analysis of Driving Factors of Grassland Degradation: A Case Study in Xilin River Basin, Inner Mongolia

**DOI:** 10.1100/2012/169724

**Published:** 2012-04-24

**Authors:** Yichun Xie, Zongyao Sha

**Affiliations:** ^1^Department of Geography and Geology, Eastern Michigan University, Ypsilanti, MI 48197, USA; ^2^International School of Software, Wuhan University, Wuhan 430079, China

## Abstract

Current literature suggests that grassland degradation occurs in areas with poor soil conditions or noticeable environmental changes and is often a result of overgrazing or human disturbances. However, these views are questioned in our analyses. Based on the analysis of satellite vegetation maps from 1984, 1998, and 2004 for the Xilin River Basin, Inner Mongolia, China, and binary logistic regression (BLR) analysis, we observe the following: (1) grassland degradation is positively correlated with the growth density of climax communities; (2) our findings do not support a common notion that a decrease of biological productivity is a direct indicator of grassland degradation; (3) a causal relationship between grazing intensity and grassland degradation was not found; (4) degradation severity increased steadily towards roads but showed different trends near human settlements. This study found complex relationships between vegetation degradation and various microhabitat conditions, for example, elevation, slope, aspect, and proximity to water.

## 1. Introduction

Natural grasslands and savannas occupy nearly half of the terrestrial globe [1, 2] and provide important services to modern societies. However, grasslands are sensitive to changing edaphic conditions, management regimes, and climate and weather variables [3, 4]. With growing human populations and intensifying development, degradation of natural grasslands has been observed in many regions of the world and is a serious concern [5]. Therefore, understanding the factors driving grassland degradation is increasingly critical to the conservation and, in some cases, the restoration of these fragile ecosystems [6, 7]. Studies on the driving factors of grassland degradation can provide information for understanding vegetation deterioration pathways and, thus, maintain ecosystem functioning and services. Heras et al. [8] identified soil quality, revegetation treatments, and climatic conditions as main driving forces in a Mediterranean dry environment. There is a need for adopting proactive grassland conservation measures and for forecasting vegetation responses to future environmental changes [9]. It is essential for policy makers to understand how vegetation responses to environmental and social changes. However, due to our limited understanding of these socioecological systems when identifying potential drivers and their possible vegetation responses, policy initiatives aimed at sustainability in vegetation ecology may fail [10].

Grassland degradation may be a complex collection of dynamic processes (e.g., desertification, salinisation, soil compaction, soil water-logging, wind erosion, water erosion, etc.) [11, 12]. Evaluation of grassland health involves assessing a large number of ecological attributes with a set of well-defined indicators, which are usually difficult or costly to measure [13]. One of these indicators is the “state” of grassland health [14]. A state usually includes one or more different biological (including soil) communities that occur on a particular ecological site and have three attributes (soil/site stability, hydrologic function, and biotic integrity) [13]. For instance, a state may include different plant communities that are connected by community pathways [15, 16]. Changes between states are referred to as “transitions.” Unlike community pathways, these “threshold” transitions are not reversible by simply altering the intensity or direction of factors that produced the changes [13]. Different patterns of vegetation transitions may reflect different stages of ecosystem stability [7, 17]. Based on the studies of the relations between ecosystem structure, function, degradation, restoration, and transition, Cortina et al. [18] confirmed that those aspects of an ecosystem were related to each other. A plant community transition is often regarded as an indicator of grassland degradation. Few studies, however, have attempted to examine vegetation transitions with the purpose of land restoration [19].

It is of practical interest to underpin causal relations between vegetation degradation (transition) and their driving factors. Several studies developed plant functional-type-based models to explore physical and biological mechanisms between vegetation transitions and environments [20, 21]. Others used empirical models to investigate the causes of vegetation degradation [22–24]. For example, Zhao et al. [25] presented a composite index of VWR (vegetation water ratio), combining land surface water index and enhanced vegetation index, to facilitate an identification of vegetation transitions by simply comparing the values of VWR at different stages. It is noted that natural processes rarely cause vegetation transitions, which are often induced by human disturbances. In many cases, natural processes are intensified by social factors, leading to vegetation degradation. Hence social sciences should be integrated into these plant functional models or empirical models in order to construct more effective models to study grassland degradation [24]. There is an urgent need to build causal diagrams of human-nature interactions through interdisciplinary collaboration [26].

Based on literature reviews, five groups of factors are identified to induce grassland degradation to noticeable degrees. The first group is the biophysical variables, including those reflecting global climate change [27]. The second group includes the botanic (or biotic) variables. Among these variables are plant cover and plant productivity. The Normalized Difference Vegetation Index (hereafter NDVI) provides a measure of the greenness of vegetation. NDVI or its derived forms mayindicate the productivity of vegetation [25]. The third group of variables deals with the impacts of livestock and wildlife. Increased grazing intensity is the most significant driving force of grassland degradation [28]. The fourth group of variables describes socioeconomic development and human interferences on grassland [29]. Land degradation in the dry-lands is mainly expressed by a reduction of biomass productivity, and it is also a manifestation of unsustainable development often associated with poverty [30]. Finally local habitat conditions, such as, water accessibility, elevation, slope, and slope aspect, may affect grassland degradation.

It is worth pointing out that evaluating the driving forces of change and projecting future changes requires a commitment to methodological pluralism and critical interpretation of social and environmental data [31]. For instance, studies of causal relationships between grassland transitions and their driving factors were confronted with several challenges. First, frequent or continuous vegetative time series data are needed to detect vegetation transition patterns [25]. Second, socioeconomic data synchronous with the data of vegetation transitions rarely exist. Third, due to the limitation of data availability, studies on grassland degradation through vegetation transitions have been mostly done on a plot basis [32]. Plot-based studies rely heavily on field surveys, which are too time-consuming and costly to conduct for large areas, although they are effective for obtaining accurate vegetative data. However, new techniques for vegetation data collection and mapping raise the possibility of quantifying grassland properties remotely [33, 34]. Satellite platforms, in particular, offer an effective means of collecting contemporary data over vast areas and in short periods of times [35, 36]. The approaches based on remotely sensed data have been increasingly applied in vegetation transition studies [25, 37].

Here we describe a systematic approach for applying a Binary Logistic Regression (BLR) model to explore grassland degradation and its driving factors in the Xilin River Basin of Inner Mongolia, China—representative of the world's largest contiguous terrestrial biome, the Eurasian steppe [38, 39].  Section 2 describes the data and analysis methodology. The results of our analysis are provided in Section 3. Finally, our findings and conclusions regarding vegetation degradation are discussed in Section 4.

## 2. Study Area, Data, and Methods

### 2.1. Study Area

**Figure 1:**
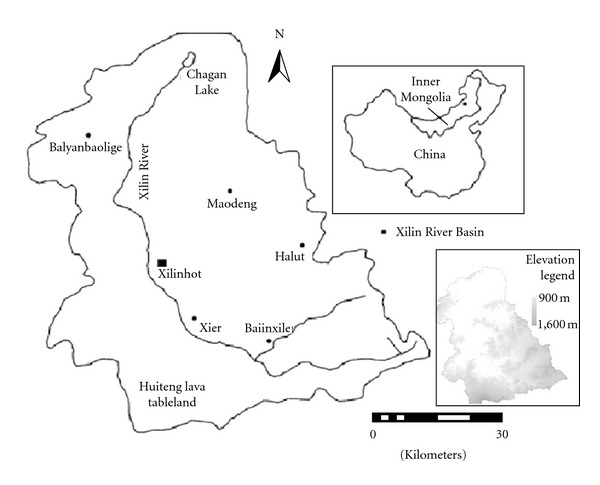
The research region.

The Xilingol River Basin, situated between 43°26′ and 44°29′ North and 115°32′ and 117°12′ East, is representative of the vast steppe of northern China [40]. More than 90% of the land in the region is covered by grassland [5]. From southeast to northeast, the altitude gradually decreases from 1608 to 902 meters above sea level (Figure 1). The basin's total area is about 10,000 square kilometers (km^2^) and has an average annual temperature range from 1 to 2°C [41]. Annual mean precipitation is around 300 mm, 60–80% of which occurs between June and August, coinciding with the highest temperatures (May to September) [5, 42, 43]. The whole region is divided into 27 administrative units, including a Xilinhot urban area, 4 pastures, and 22 villages. Herd husbandry provides the main income for local farmers. Over the last several decades, the human population has grown and overgrazing is now an important concern for local governments and ecologists due to its vegetation degradation. Previous studies and onsite surveys showed that continuous overgrazing imposes a severe threat to the sustainability of this grassland.

The study region includes 11 vegetation communities (as indicated by climax species) [44]. Two climax vegetation communities, that is, *Stipa grandis* (SG) and *Leymus chinensis* (LC), are widely distributed over the study area and consist of the local reference states [38, 45]. It has been confirmed through field survey that SG and LC communities were partially replaced by degraded vegetation communities within the past two decades, most of which are *Cleistogenes squarrosa* (CS) and *Artemisia frigida* (AF) [39]. For example, on the Xier Plain, located at the middle to the upper reach of the Xilin River, AF and CS almost dominate the area, where the primary climax vegetation was supposed to be SG and LC, indicating a significant vegetation transition. The transition of either SG or LC communities to any other vegetation community is defined as grassland degradation in this study.

### 2.2. The Data Sources and Analysis Methods

Four binary logistic regression models were constructed and the dependent variables of grassland degradation were described as transitions of LC and AG to any other vegetation communities within two periods, 1985–1998 and 1998–2004 (Table 1(a)). Two sets of nine independent variables from two years (1985 and 1998) were obtained (Table 1(b)). The first two independent variables indicating the biotic conditions are the density of the base vegetation (DV) and the normalized difference vegetation index (NDVI). The third independent variable is the average grazing intensity (AGI), reflecting the impact of livestock. Two other independent variables, the distance to road (DR) networks and the distance to settlement (DS) centers, are intended to reflect human disturbances. Finally the local habitat conditions are described by four variables, elevation (ALT), slope (SLP), slope orientation (ORI), and the distance to water (river) body (DW).

**Figure 2:**
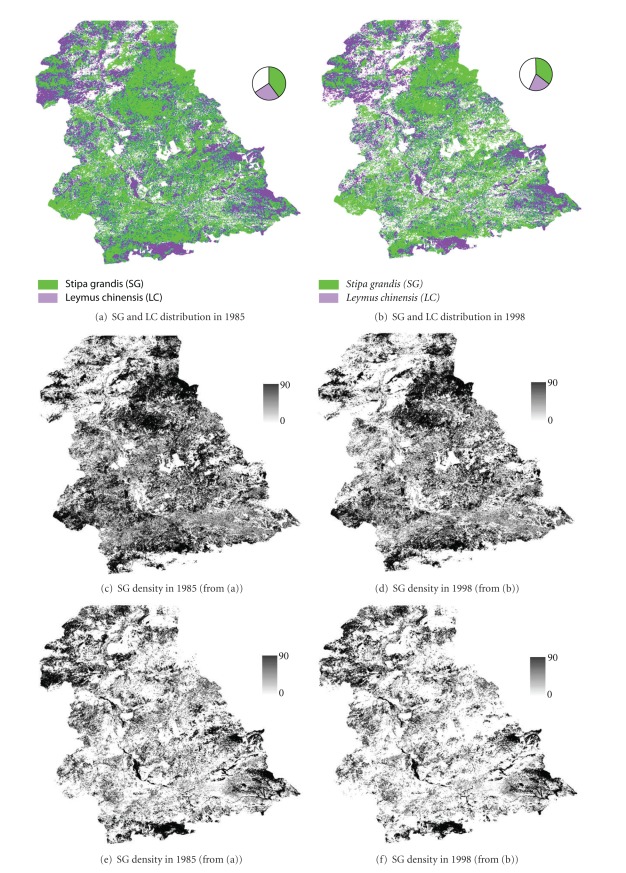
The base vegetation community maps and the density of vegetation communities.

**Figure 3:**
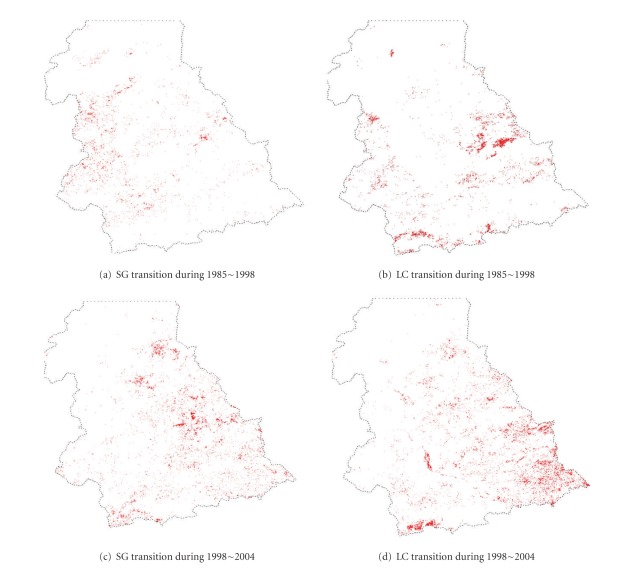
Spatial distributions of vegetation successions of SG and LC. Red: occurrence of vegetation transitions; white: nonoccurrence of vegetation transitions.

The four dependent variables of grassland degradation and two independent variables (DV and NDVI) are obtained from Landsat ETM+ and TM image processing. Landsat ETM+ images were used to classify the vegetation communities in 2004 [44], while Landsat TM images were processed to create the vegetation cover maps in 1985 and 1998 [46]. Therefore, three vegetation cover maps at an almost identical date in different years (1985, 1998, and 2004) were produced and the spatial distributions of SG and LC were mapped (Figures 2(a) and 2(b)). The dependent variables are generated through the change detection analysis. They are binary, 1 indicating transitions of LC or AG to any other degraded vegetation communities in two periods, 1985–1998 and 1998–2004, and 0 denoting no transitions (Figure 3). 

**Figure 4:**
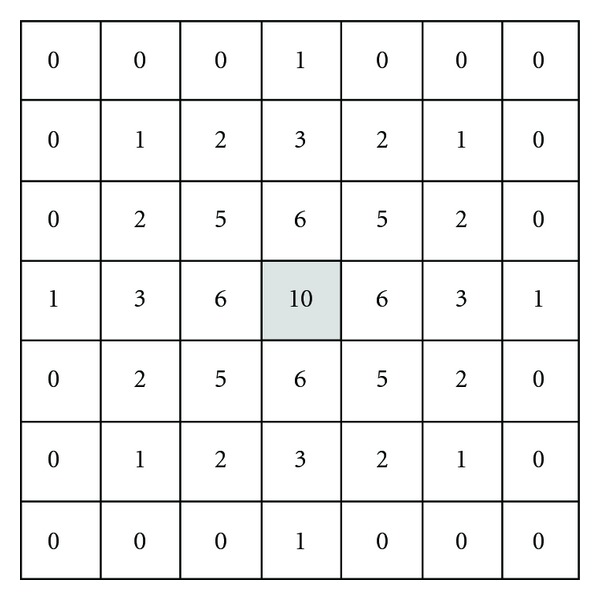
Filter kernel for density mapping.

The density of base vegetation (DV) was computed on the basis of the spatial distribution of SG and LC (of 1985 for succession between 1985 and 1998, and of 1998 between1998 and 2004). A 7 × 7 density kernel (Figure 4) is first applied to resample SG and LC (denoted by DK used in (1)) over the study region, which ensures a continuous interpolation of the density of the vegetation communities. This kernel places more weight on the central pixel but assigns less weight to adjacent pixels according to Tobler's first law of geography “things that are closer are more alike” [47, 48]. 

The density function is defined as

(1) $${\text{DV}}_{ij}=\overset{i+3}{\underset{i^{\prime }=i-3}{\sum }}\;\overset{j+3}{\underset{j^{\prime }=j-3}{\sum }}\left(P_{i^{\prime }j^{\prime }}\times {\text{DK}}_{i^{\prime }j^{\prime }}\right),$$

where DV_*ij*_ is the *DV* value for the current pixel (*i*, *j*) in the density image vegetation communities (SG or LC), *i*′ and *j*′ are the relative coordinate locations of the pixel, *P*_*i*′*j*′_ is a binary DV value (1 when the pixel is covered by the base vegetation type (SG or LC) and 0 when covered by other types of vegetation communities) of the pixel located at *i*′ and *j*′, and DK_*i*′*j*′_ is the kernel density value. The relative value of DV_*ij*_, which has a minimum value of 0 and maximum 90, takes into consideration the nearby vegetation communities. When it equals the maximum value of 90, it indicates that all pixels within the sampling window are SG (or LC). On the contrary, the minimum value of 0 indicates that all pixels within the window are filled with other vegetation types. Generally, the larger the value is, the greater the chance is that the vegetation community is SG or LC. Therefore, the maps produced by (1) are referred to as the base vegetation cover density. Since there are the two periods (1985~1998 and 1998~2004) and two vegetation communities considered, 4 density distribution maps (DV_SG-1985_, DV_SG-1998_, DV_LC-1985_, and DV_LC-1998_) can be produced, which are shown in Figures 2(c), 2(d), 2(e), and 2(f), respectively. 

NDVI is an indicator of vegetative greenness. The use of NDVI in studying vegetation changes has a long history in large area ecological research [25]. NDVI is calculated using a near infrared and red spectral band from Landsat TM data for 1985 and 1998, respectively:

(2) $$\text{NDVI}=\frac{\text{NIR}-\text{RED}}{\text{NIR}+\text{RED}}.$$

The average grazing intensity (AGI) is computed based on an extensive field survey conducted in 2004. The statistical data of grazing intensity at the village level on a yearly basis (from 1985 to 2004) were systematically collected from the local village committees. There are five recorded animal species, that is, sheep, horse, buffalo, camel, and donkey. Because different animals have varied impacts on grass consumption and vegetation damage, each of the animals is converted to a standard unit, the sheep unit, to compute the grazing intensity. Based on the survey with the local herdsmen, an average grazing intensity (AGI) is calculated on the basis of the following equation:

(3) $$\text{AGI}=\overset{5}{\underset{i=1}{\sum }}C_{i}\times N_{i},$$

where *C*_*i*_ is the coefficient for animal *i*, and *N*_*i*_ is the total number of the animal. To determine each coefficient of *C*_*i*_, a questionnaire survey was done and the answers from the local farmers were synthesized to compute the coefficient. Specifically, one horse is equivalent to 6 sheep and thus is multiplied by 6 to transform into the sheep unit. From the same survey, cattle, camel, and donkey are multiplied by 5, 7, and 3, respectively. As a result, the average AGI during the period of 1985 to 1998 is noted as AGI_(85–98)_. Similarly, AGI_(98–04)_ is the averaged value of AGIs from 1998 to 2004. As shown in Table 1, the average grazing intensity during 1998~2004 almost doubles that during 1985~1998. 

The road network is mainly derived from the topographic data and local road maps, with quality checking using the TM and ETM+ images. The road network was also checked during the field survey in 2004. Settlement centers and water bodies (rivers) were derived from a similar approach as the road network. The ERDAS analysis function “SEARCH” was applied to create the three maps for DR, DS, and DW, respectively, with three vector layers showing the road network, water bodies (river), and village settlement centers. 

The remaining independent variables reflecting local habitat conditions were computed from a digital elevation model (DEM) at a scale of 1 : 25,000. The DEM was resampled to create a new DEM at the spatial resolution of 30 × 30 m so that the new DEM had the same spatial resolution as the vegetation community maps. This resampled DEM was then used to generate three topographical maps of elevation (ALT), slope (SLP), and slope orientation (ORI). Considering the sensitivity of vegetation growth in certain sunshine directions, the original aspect value (OLD_ORI_) derived from DEM is further orientated with the following equation:

(4) $${\text{NEW}}_{\text{ORI}}=360-{\text{OLD}}_{\text{ORI}},\;\text{if}\;{\text{OLD}}_{\text{ORI}}>180.$$

This transformation (4) indicates that the incident sun direction was an important factor for green grass growing and thus provides a better indicator to impact vegetation transition than the original aspect. 

### 2.3. Binary Logistic Regression Model

Binary Logistic Regression (BLR) is a type of predictive modeling that tries to predict the binary probability of an outcome, for example, the occurrence or nonoccurrence of an event. In this study, BLR model was applied to fit the relationships between the dependent variable, the occurrence of vegetation degradation at any location (pixel *i*), and the independent variables listed in Table 1. A total of 5,000 samples labeled with one of two climax vegetation communities (SG or LC) were randomly chosen independently for each model. For example, let us take 5000 pixels labeled as “SG” in the year of 1985 as shown in Figure 2(a) to analyze SG degradation during 1985~1998. Four fifth out of all the selected samples were randomly selected to build a BLR model while the rest were kept for validation. The probability of having vegetation degradation (or SG transition to another vegetation type) at any location (pixel) was estimated by

(5) $$\ln\left[\frac{p\left(y=1\;|\;X\right)}{1-p\left(y=1\;|\;X\right)\;}\right]=\beta_{0}+\overset{n}{\underset{i=1}{\sum }}\beta_{i}\chi_{i},$$

where *p* means the probability of occurrence (*y*), that is, the probability of having SG transition during 1985–1998. 1 − *p* is the opposite probability, that is, nonoccurrence of SG transition. *x*_*i*_ is the independent variables. *β*_0_ and *β*_*i*_  (*i* = 1,2,…, *n*) are the estimated parameters, and *p*(*y* = 1 | *X*) is the probability that *y* takes the value 1, given the vector of independent variables *X*. The quantity *p*(*y* = 1 | *X*)/(1 − *p*(*y* = 1 | *X*)) is referred to as the odds, whereas ln⁡[*p*(*y* = 1 | *X*)/(1 − *p*(*y* = 1 | *X*))] is called the logit. 

The same approach has been taken for the SG transition during 1998–2004, as well as the LC transitions during 1985–1998 and 1998–2004. Therefore, four BLR models of vegetation degradation were obtained. In addition, two more tries of building these BLR models were tested to validate the applicability of these models by introducing two additional sets of 5000 randomly selected samples from each base vegetation map and for each model, respectively. Similar results in terms of the coefficients (*β*_*i*_, *i* = 0,1, 2,…, *n*) in the models and the prediction accuracies of the models were obtained. Little variation (within 5.0%) was noticed among all of the three trials in terms of the value of each of the coefficients and the overall prediction accuracy. 

The unstandardized logit coefficient, that is, the *β* in (5) measures the absolute contribution of a variable in determining the probability that a particular vegetation degradation occurred. However, this information may be misleading when the unit adopted is not consistent from variable to variable as a result of disparities in units and scales of measurement. Thus, prior to performing a logistic regression, we standardized the independent variables with zero mean and the unit standard deviation, using the formula:

(6) $$x_{i}^{\prime }=\frac{x_{i}-{\bar{x}}_{i}}{\sigma_{x}},$$

where *x*_*i*_′ is the standardized value of a variable, *x*_*i*_ the value of the original variable, ${\bar{x}}_{i}$ the mean, and *σ*_*x*_ the standard deviation of the original variable. 

After substituting *x*_*i*_ with *x*_*i*_′ in (5), the result of the regression can be expressed in terms of conditional probability at any spatial location (pixel *i*) to be predicted:

(7) $$p_{i}=\frac{\exp\left(\beta_{0}+\sum_{i=1}^{n}\beta_{i}\chi_{i}\right)}{\left(1+\exp\left(\beta_{0}+\sum_{i=1}^{n}\beta_{i}\chi_{i}\right)\right)}.$$

The probability *p*_*i*_ of vegetation degradation at a location *i* in the study region could be calculated according to (7). It is a straightforward computation based on (7) to make a probability map of vegetation degradation over a given region when the required variables for the model are available. 

## 3. The Results

Regression models were run using the SPSS forward likelihood ratio (LR) method (see http://www.ats.ucla.edu/stat/spss/examples/alda/default.htm). The relative contributions of the independent variables to vegetation degradation are expressed by the “odds ratio”, exp⁡(*β*). An odds ratio greater than 1 indicates a positive effect. In other words, the odds of vegetation degradation increase by 1 standard unit with a unit increase in an independent variable. An odds ratio smaller than 1 indicates a negative relationship, which means that an increase in the independent variable decreases the odds of vegetation degradation, whereas an odds ratio of 1 indicates that the odds of vegetation degradation is neutral to an increase in the independent variable. 

### 3.1. Spatial and Temporal Patterns of Vegetation Transitions

Two dominant vegetation communities, *Stipa grandis* (SG) and *Leymus chinensis* (LC), are widely distributed in the study area as reflected on the base vegetation community maps shown in Figures 2(a) and 2(b). Statistical analyses of these two maps revealed that SG or LC amounted to more than 50% of the total area. Patches labeled with LC occupied 25.9% in 1985 and 22.0% in 1998, while SG displays even wider distributions, 39.9% in 1985 and 34.9% in 1998. From 1985 to 1998, the areas covered by LC and SG all decreased by 3.9% and 5.0%, respectively. Four density maps (Figures 2(c), 2(d), 2(e), and 2(f)) are derived from Figures 2(a) and 2(b). The density maps of LC and SG showed a slightly decrease in 1998 (Figures 2(d) and 2(f)) when compared with the density maps in 1985 (Figures 2(c) and 2(e)), which demonstrated a certain degree of vegetation degradation from either SG or LC to other vegetation communities over this study period. 

Based on the vegetation cover recorded for three dates (1985, 1998, and 2004), actual vegetation transition maps were produced (Figure 3). Visual interpretation of the spatial distribution of the vegetation transitions showed that transitions were not evenly distributed over the study area and that significant transitions occurred at the middle or southern part of the region. Though this distribution might be correlated with the distribution of the base vegetation communities (SG and LC), other factors need to be further examined to understand how they affect the vegetation degradation process. 

### 3.2. Driven Factors of Vegetation Successions

On the basis of the significance levels of the model coefficients and Goodness-of-Fit tests (Pr > Chi-square is greater than 0.05 for all cases) (Tables 2 and 3), the BLR models performed well explaining the probability of vegetation transition, and their fitted models showed moderate predicting accuracy (over 75.0% for all models, Table 4). One of the biggest contributors to the vegetation transition is the density of base vegetation communities (DV_SG_ and DV_LC_). DV_SG_ and DV_LC_ in both periods (1985–1998 and 1998–2004) showed significantly positive correlations with the occurrence of vegetation degradation (indicated by the value of Exp(*β*), and the odds ratio in Tables 2 and 3). This finding is somewhat opposite to the findings about grassland degradation conducted by others, which suggest that degradation often happens in rangeland of poor health [49]. 

Another factor picked up by the BLR models is the grazing intensity. It is commonly reported in current literature that the increase of grazing intensity leads to intensified grassland degradation [50, 51] and decreased ANPP and species richness [52]. Although three out of four BLR models reached a similar finding, the SG transition during the period of 1985~1998 showed a negative relationship with the increase of grazing intensity. In other words, it could be interpreted that SG showed less degradation during 1985–1998 when the grazing intensity increased (Table 2(a), Exp(*β*) = 0.976). Our analysis points out that there exist complex relationships between grazing intensity and grassland degradation. As can be seen from the statistical result of the data (Table 1), the grazing intensity over the study region during 1998~2004 (averaged 120.7 sheep unit/km^2^) nearly doubled that during 1985~1998 (averaged 74.3 sheep unit/km^2^). SG was more resilient to degradation when the grazing level was low while LC was more vulnerable to the intensification of grazing. The probability of the LC degradation increased by 1.12 and 1.10 over both periods (1985~1998 and 1998~2004), respectively (Table 3). Our finding also suggests that the average grazing intensity (AGI) at current level could have exceeded the carrying capability of the grassland ecosystems, as the degradation is a common phenomenon over the entire study area at present. 

The contribution that the road network makes to the vegetation degradation can be seen from Tables 2 and 3. The density of the road network had a significant impact to the degradation of both SG and LC over the periods of 1985~1998 and 1998~2004. On average, an increase of the distance to the nearby road by 1 distance unit (i.e., 1.4 km in 1985~1998 and 1.2 km in 1998~2004, see Table 1) decreases the odds of SG degradation by a factor of 0.89 and 0.80 during the two periods, respectively. Similarly, an increase of the distance to the nearby road network by 1 distance unit decreases the odds of SG degradation by a factor of 0.80 and 0.87, respectively. 

The variable of DS is an indicator that describes possible impact of human disturbance on vegetation degradation. DS was negatively related to the probability of both SG and LC degradations during the period of 1985~1998 (Tables 2 and 3). An increase of DS by 1 distance unit (i.e., 3.5 km) decreased the odds of SG and LC degradation by 11% and 22% during this period, respectively. However, during the other period of 1998~2004, DS was excluded by BLR models, indicating that the distance to DS was no longer a significant factor for predicting the probability of vegetation degradation. It was reported that human activities had a complicated impact on vegetation dynamics, depending on spatial and temporal scales at which assessments were conducted [53]. In addition, it was confirmed through our field survey that many settlement centers appeared during 1998~2004 due to rapid socioeconomic growth in the study region. Many of these newly developed settlement centers were not primarily dependent on grazing. Therefore, the impact of DSs on grassland degradation was significantly reduced during the second study period. 

The impacts of the topographical factors on grassland degradation showed complex patterns. Elevation was the sole factor that significantly correlated with the occurrence of vegetation degradation based on the BLR models. High degradation probabilities of both SG and LC took place largely in the areas with high elevations. In the middle and upper reaches of Xilin river (south-east part) where the elevations are higher than other places, vegetation degradations were widely noticed (Figure 3). However, the other topographical factors of aspect and slope were excluded by all BLR models. Our null hypothesis that the topographical factors, aspect and slope, might affect the degradation occurrence was rejected by the result. 

Validation of the above models is given in Table 4. For the selected sites used to build the models, the overall accuracies for SG and LC degradation are 84.5% and 82.7% during 1985~1998, and 85.2% and 83.8% during 1998~2004, respectively. For the validation sites, the overall accuracies for SG and LC degradation are a little lower, which is 75.5% and 78.3% during 1985~1998, and 77.6% and 78.4% during 1998~2004. The results demonstrate that the fit models can generally be used to predict vegetation degradation over the study area with a moderate prediction accuracy (over 75%). 

**Figure 5:**
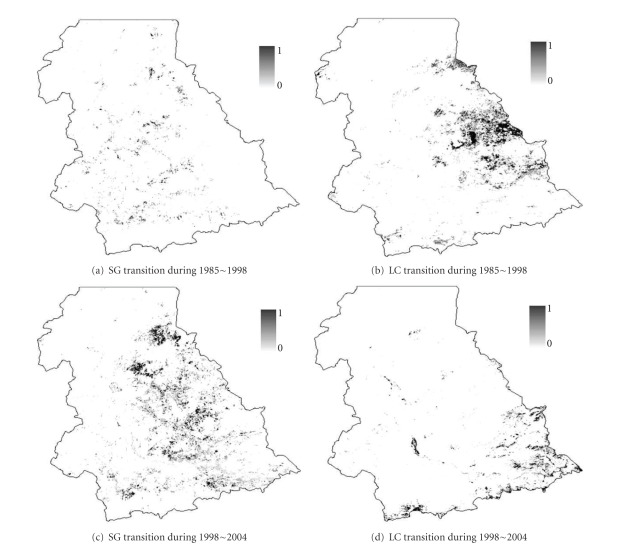
Probability mapping of vegetation transitions.

Based on the BLR models, four maps showing the probabilities of the vegetation degradation (i.e., SG and LC degradations during 1985~1998 and 1998~2004) are produced. The pixel value in the maps shows the modeled probability of vegetation degradation over the study area (Figure 5). When compared with the observed incidences of vegetation degradation (Figure 3), the probability maps have assigned relatively high probability values to the locations where the vegetation degradations were actually observed, indicating that the fit models were useful for predicting vegetation degradation. With an increasingly improved access to latest natural and socioeconomic data, it is feasible to update these degradation maps for the purpose of prediction and for best practices of grassland (ecological) management.

## 4. The Discussion and Conclusion

Grassland degradation has been regarded as an important indicator of grassland ecosystem health. However, there are limited researches dealing with the relationship between vegetation degradation and its driving factors (both natural and socioeconomic) that may influence the degradation pathways. Researches on grassland degradation have a twofold practical application, identifying possible driven forces that cause and accelerate the degradation processes, and providing scientific data for making informed decisions in order to change the degradation pathways in the direction of keeping grassland ecology sustainable.

### 4.1. How Vegetation Degradation Responds to Various Driving Factors

There are four important findings regarding the responses of vegetation degradation to various driving factors. First, grassland degradation has shown a strong positive correlation with high density or productivity of climax communities. This finding might be contradictory to natural ecological processes but is a tragic outcome of human greed of seeking maximum profit. Under the eager of catching up with economic booms and enjoying material wealth, herdsmen made their best bets in most productive grassland in order to have maximum investment returns. Second, the decrease of biological productivity (NDVI in our case study) cannot be simply regarded as a direct indicator of grassland degradation. NDVI showed no significant relationship with vegetation degradation in this study. Similar studies on vegetation degradation did show that the indexes (NDVI and other forms) derived from remotely sensed imagery exhibited significant spatial and temporal correlations with vegetation degradation [54]. The different finding from our research was largely attributed to the fact that heavy (or excessive) grazing and subsequent degradation occurred in climax plant communities with high productivity. 

Third, a causal relationship between grazing intensity and grassland degradation only holds when grazing intensity reaches a certain threshold (or a balance point). This threshold also varies with different plant communities. Under this threshold of grazing pressure (e.g., SG during 1985~1998), SG was resilient to degradation. However, due to the lack of yearly data and longitudinal data (before 1985), we were not able to identify the threshold values of grazing intensities for SG or LC degradation. It will be an important piece of future research to establish grazing intensity threshold values for various plant communities so that grassland strategic conservation plans could be made on the basis of scientific data about grazing intensity or grassland bearing capacity. 

Fourth, complicated relationships have been found between grassland degradation and direct human disturbances. The development of road network was a leading cause of grassland degradation, which was consistent with our field observations. Degradation severity increased steadily towards road network. However the impact of human settlements on grassland degradation showed a clear temporal change. Earlier settlements served primarily for animal husbandry and were closely linked grazing activities and thus had significant impact on grassland degradation. Newly village centers and towns have played more roles as economic or urban centers and their direct impacts on grazing have been reduced. 

### 4.2. Policy Implications for Preventing Vegetation Degradation in the Study Region

Overgrazing occurred widely in the study area. With this in mind, there have been areas of fenced grazing, which were excluded in our case study. It is believed that such practices could be expanded over larger areas as an effective measure to protect grassland vegetation and prevent degradation [55]. Another phenomenon is the fast development of the unplanned road network. As evidenced in our field investigation, wherever a road was extended, the grassland was destroyed adjacent to the road. On the other hand, a road, if well planned, could not only bring more convenience to the local population but also be ecologically beneficial because roads extended to remote areas allow the rapid movement of animals to balance grazing intensity over a large region. This may reduce overgrazing in one area while other areas remain ungrazed. Unfortunately, because of immediate economical incentives, the development of roads was unevenly distributed, making vegetation degradation even worse since these were concentrated near town or residential centers, or current husbandry centers where severe degradation (vegetation degradation) had already occurred. Therefore, more attention should be given to protect vegetation communities near the areas along roads, for example, establishing vegetation conservation zones, and to construct road networks in remote areas. 

Human disturbance, mainly indicated by the variable DS, is an important factor that stimulated vegetation degradation in the period of 1985~1998. However, this phenomenon was not obvious in the later period, 1998~2004, even when more settlement centers were established. The difference of DS impact upon vegetation degradation is attributed to better vegetation protection measures near villages in the second period. Therefore, a policy of protecting vegetation patches that are under severe degradation around older settlements is effective. The implication of this policy is that a rigorous effort should be made to limit unplanned expansions of new settlements. 

### 4.3. Selection of the Model Variables

The selection of the candidate variables for modeling the grassland vegetation degradation has been guided by our literature review, our field survey, and the characteristics of our study area. Many studies have proven that the changes in the global natural environment have made significant impact on vegetation dynamics [56–58]. The critical changes in natural environment are typically exemplified by global climate deteriorations, among which are the rising temperature and the shortage of precipitation in grassland areas [59]. The climate changes were regrettably not taken into consideration in our current study due to the fact that the study area covers a total area of only 10,000 km^2^, which strides about 100 km in longitude and 100 km in latitude. The climate data collected at a few weather stations over the study area showed little variations. 

## 5. Conclusions

Vegetation degradation is an important indicator of grassland ecosystem health. In this study, we examined the relationship between the occurrences of vegetation degradation and its driving factors in the Xilin River Basin, Inner Mongolia, China. As *Stipa grandis* and *Leymus chinensis *are the two most dominant grassland communities in the study area, their degradation patterns have important implications in terms of grassland ecology and management. Binary logistic regression (BLR) was used to fit the nonlinear correlations between the occurrences of vegetation degradation (dependent variable) and nine independent variables over two consecutive periods, 1985~1998 and 1998~2004. The independent variables include two indicating biotic conditions (the density of base vegetation and the normalized difference vegetation index), three reflecting human interferences (average grazing intensity, the distance to road network, and the distance to settlement center), and four denoting local habitat conditions elevation, slope, slope orientation, and the distance to water (river) body. 

 Several important findings were suggested by the BLR model. First, four variables, including the base vegetation density (for SG and LC), the average grazing intensity, the distance to road network, and the altitude, were important determinants of grassland degradation for both plant communities (SG and LC) and over both study periods. Secondly, some of our findings provide new insights into the causes of grassland degradation. For instance, severe degradation often happens in the most productive grassland; grassland is in general resilient to degradation when grazing intensity is kept under a certain threshold; the construction of road networks is the most destructive factor causing grassland degradation; the negative impact of human settlement on grassland degradation is much significant when the residents are primarily herdsmen. These findings should have clear policy implications in grassland management. Thirdly, the BLR models have moderate accuracy levels, over 75%, indicating that the BLR models are acceptable in studying grassland vegetation degradation, but some caution should be exercised by investigator. The models adopted in the current study should also work for other grassland regions when required data are available. 

Finally, there are some limitations in current BLR models that should become the focus of future research topics or methods concerning grassland degradation. Due to the limitation of available data, current BLR models were implemented with periodic data (1985–1998 and 1998–2004). The results could be more convincing and accurate if the BLR models could be run with a yearly based dataset. The applicability of the BLR models could be extended to a regional scale if such variables as those which can tell spatial variations of climate changes could be added into the BLR models.

## Figures and Tables

**Table:** (a) Variables used to fit the probability of vegetation successions by BLR

Variable abbr.	Description	Minimum	Maximum	Mean	Std. dev
^†^NDVI_85_	Normalized difference vegetation index in 1985	−0.22	0.59	0.19	0.07
^†^NDVI_98_	Normalized difference vegetation index in 1998	−0.21	0.66	0.16	0.06
^†^DV_(SG_85)_	Density of vegetation LC in 1985	0.00	90.00	52.91	29.63
^†^DV_(SG_98)_	Density of vegetation LC in 1998	0.00	90.00	66.01	20.62
^†^DV_(LC_85)_	Density of vegetation SG in 1985	0.00	90.00	12.62	21.59
^†^DV_(LC_98)_	Density of vegetation SG in 1998	0.00	80.00	5.84	11.14
^‡^DS (km)	Distance to village settlement center	0.00	8.10	3.53	0.59
^‡^DR_85_ (km)	Density of road in 1985	0.00	2.10	1.40	1.22
^‡^DR_98_ (km)	Density of road in 1998	0.00	2.05	1.17	1.18
^‡^DW (km)	Distance to water (river) body	0.00	2.55	0.87	0.73
^♀^SLP (degree)	Slope in degree	0.00	66.00	4.79	6.30
^♀^ORI (degree)	Orientation from North in degree	0.00	180.00	76.46	59.00
^♀^ALT (m)	Altitude in meters	902.00	1608.00	1160.81	73.91
^*♯*^AGI_(85–98)_ (sheep/km^2^)	Average grazing intensity during 1985~1998	25.95	92.57	74.32	23.69
^*♯*^AGI_(98–04) _ (sheep/km^2^)	Average grazing intensity during 1998~2004	79.28	170.12	120.69	18.88

**Table:** (b) Dependent variable (vegetation succession) description

Case no.	Succession Type	Period	Description
1	SG to CS/AF	1985~1998	Vegetation succession from SG to CS/AF during 1985~1998
2	SG to CS/AF	1998~2004	Vegetation succession from SG to CS/AF during 1998~2004
3	LC to CS/AF	1985~1998	Vegetation succession from LC to CS/AF during 1985~1998
4	LC to CS/AF	1998~2004	Vegetation succession from LC to CS/AF during 1998~2004

Data extracted from ^†^: Landsat imagery and the ground truthing is referred to Xie et al. [46] and Sha et al. [44]; ^♀^: digital elevation model; ^‡^: road network map, annual economic statistics of the local governments and Landsat imagery; ^*♯*^: annual economic statistics of the local governments.

**Table 2:** Results of BLR analysis for SG succession.

Variable	*β*	S.E. (*β*)	Sig.	Exp (*β*)	95.0% C.I. for Exp(*β*)	
					Lower	Upper
(a) During 1985~1998						
DV_SG-85_	.022	.003	<.001	1.017	1.017	1.028
AGI_8–98_	−.016	.004	<.001	.976	.976	.993
DR_85_	−.115	.012	<.001	.891	.832	.945
ALT	.122	.021	<.001	1.130	1.116	1.214
DS	−0.109	0.23	<.001	0.890	0.877	0.913
*SLP						
*ORI						
*NDVI_85_						
*DW						
Intercept	−1.912	.706	<.001	.148		

(b) During 1998~2004						
DV_SG-98_	.040	.003	<.001	1.041	1.036	1.046
AGI_(9–04)_	.109	.002	.031	1.115	1.000	1.208
DR_98_	−.161	.021	<.001	.851	.800	.889
ALT	.191	.019	<.001	1.210	1.112	1.277
*SLP						
*ORI						
*NDVI_98_						
*DW						
*DS						
Intercept	−14.411	.853	<.001	.000		

Hosmer and Lemeshow Goodness-of-Fit Test: Chi-square = 1.802, Pr > Chi-square = .213						

*n* = 4000.

Maximum likelihood estimate of the parameter. S.E. (*β*): estimated standard error of the parameter estimate; Wald *χ* ^2^: Wald chi-squared statistic; Sig.: *P* value of the Wald chi-squared statistic; Exp(*β*): odd ratio.

*variables excluded by the logistic regression model after the run.

C.I.: confidence intervals.

The cut value is 500.

**Table 3:** Results of BLR analysis for LC succession.

Variable	*β*	S.E. (*β*)	Sig.	Exp (*β*)	95.0% C.I. for Exp(*β*)	
					Lower	Upper
(a) LC succession: during 1985~1998						
DV_LC-85_	0.040	0.003	<0.001	1.041	1.035	1.046
AGI_(85–98)_	0.013	0.005	0.005	1.013	1.004	1.022
DR_85_	−0.123	0.021	0.007	0.884	0.801	0.923
ALT	0.006	0.001	<0.001	1.006	1.005	1.008
DS	−0.201	0.027	<0.001	0.818	0.779	0.900
*SLP						
*ORI						
*NDVI_85_						
*DW						
Intercept	−13.830	1.015	<0.001	0.000		

Hosmer and Lemeshow Goodness-of-Fit Test: Chi-square = 9.712, Pr > Chi-square = 0.286						

(b) LC succession: during 1998~2004						
DV_LC-85_	0.047	0.002	<0.001	1.048	1.043	1.052
AGI_(98–04)_	0.088	.019	<0.001	1.092	1.090	1.095
DR_98_	−0.098	0.011	<0.001	0.907	0.872	0.974
ALT	0.018	0.001	<0.001	1.018	1.008	1.010
*SLP						
*ORI						
*NDVI_98_						
*DW						
*DS						
Intercept	−15.420	0.783	<0.001	0.000		

Hosmer and Lemeshow Goodness-of-Fit Test: Chi-square = 11.867, Pr > Chi-square = 0.157						

*n* = 4000.

Maximum likelihood estimate of the parameter. S.E. (*β*): estimated standard error of the parameter estimate; Wald *χ* ^2^: Wald chi-squared statistic; Sig.: *P* value of the Wald chi-squared statistic; Exp (*β*): odd ratio.

*variables excluded by the logistic regression model after the run.

C.I.: confidence intervals.

The cut value is 500.

**Table 4:** Classification test of BLR models.

Observed		Predicted					
		Fitting cases			Validation cases		
		Succession		Percentage correct	Succession		Percentage correct
		Yes	No		Yes	No	
(a) SG succession: during 1985~1998							
Succession	Yes	1812	187	90.6	411	57	87.8
	No	434	1567	78.3	188	344	64.7
Overall percentage				84.5			75.5
(b) LC succession: during 1985~1998							
Succession	Yes	1405	160	89.8	245	43	85.1
	No	534	1901	78.1	174	538	75.6
Overall percentage				82.7			78.3
(c) LC succession: during 1998~2004							
Succession	Yes	1201	134	90.0	199	50	80.0
	No	456	2209	82.9	174	577	76.8
Overall percentage				85.2			77.6
(d) SG succession: during 1998~2004							
Succession	Yes	789	144	84.6	142	33	81.1
	No	506	2561	83.5	187	642	77.8
Overall percentage				83.8			78.4

The cut value is 500.
